# Not Quite Equal Odds: Openness to Experience Moderates the Relation Between Quantity and Quality of Ideas in Divergent Production

**DOI:** 10.3389/fpsyg.2019.00355

**Published:** 2019-03-04

**Authors:** Morten Friis-Olivarius, Bo T. Christensen

**Affiliations:** ^1^Department of Marketing, Copenhagen Business School, Copenhagen, Denmark; ^2^Copenhagen Institute of Neurocreativity, Copenhagen, Denmark

**Keywords:** creativity, quantity breed quality, equal odds rules, brainstorming, individual differences

## Abstract

Since brainstorming was introduced as a technique in 1953 it has been assumed that the best way to produce good ideas is through the production of many ideas, which has later been named the equal-odds rule. However, this finding that productivity often leads to creative quality has rarely been examined in psychometric studies of creative cognition. To close this knowledge gap, we examined the relationship between individual differences in creative personality, as assessed by the personality trait openness to experience, and both the quantity and quality of ideas produced in a divergent thinking task. Across 154 graduate students we found a positive and significant relationship between creative personality and the number of ideas produced, as well as their creative value. The present results indicate that while quantity does breed quality in creative production, the effect is moderated by individual differences, specifically the personality trait Openness to Experience. As the level of Openness to Experience increases, the relation of quantity of ideas to average Creative value gradually becomes positive and significant. We discuss the possible reasons for and implications of our findings.

## Introduction

Alex Osborn, the inventor of brainstorming, began his pioneering work on developing creative problem solving techniques in 1939. At the time, Osborn was partner in an advertising company and was frustrated by the lack of quality in the ideas produced by his employees. Experimenting with group-thinking sessions he noticed that the quality and quality of the ideas were greater than those produced by individual employees and argued: “*It is almost axiomatic that quantity breeds quality in ideation. Logic and mathematics are on the side of the truth that the more ideas we produce, the more likely we are to think up some that are good*” (Osborn, 1953/1963, p. 131). Given the simple and appealing logic, this hypothesis was almost unquestionably accepted by the brainstorming literature and has become, not just the guiding principle for creative ideation, but also the primary tool for evaluating creative performance and even the effectiveness of creativity training programs (for review see Onarheim and Friis-Olivarius, 2013).

The most compelling evidence for the idea that quantity breed quality (QBQ) was put forth by Simonton after studying the relationship between “outstanding achievements” and creative productivity both across and within careers for two decades. Based on this extensive histometric research, Simonton (1997) conceptualized what is known as the “equal-odds rule,” which states that the number of successful ideas is proportional to the number of ideas generated. In other words: the more an individual produces, the more likely s/he is to stumble upon success (i.e., QBQ). The equal-odds arise from the stochastic nature of creativity, and has been observed to be approximately constant across a career. It can be deduced from the equal-odds rule that the number of ideas produced should be unrelated to the average quality of those ideas.

However, not all research has been supportive of the equal-odds rule. Even though most studies on brainstorming has focused on finding methods to increase the quantity of ideas, those that have tested the QBQ have reported mixed results of either a positive or no relationship between the production of ideas and their quality (for review see Reinig and Briggs, 2008). There are even reports of negative effects following a high quantity of idea production.

The quantity of ideas is, however, not the only variable that may influence idea quality. Research on individual differences in creative personality has found that subjective ratings of the quality of creative ideas correlate positively with the trait openness to experience (e.g., Silvia et al., 2008; Batey et al., 2010). Openness to experience has consistently been associated with trait creativity, specifically the ability to produce a high quantity of ideas (McCrae, 1987), their quality (Silvia et al., 2008, 2009) and with achievement creativity (King et al., 1996; Nusbaum and Silvia, 2011).

This opens the theoretically important, but not yet tested, possibility that the QBQ relation may be moderated by individual differences in creative personality. In so far as such a potential moderation effect can be documented, it would help further develop theoretical models of creative production such as the QBQ and Equal-Odds frameworks, speaking to the need of incorporating individual differences as moderators.

It is important to note that almost all of the work that has been done on the equal-odds rule, whether defining or evaluating it, has been based on data from high achieving individuals. A possible explanation for the mixed QBQ results is therefore an inattention to the possibility that the QBQ and equal-odds rule may not apply equally to individuals with varying levels of creativity. We therefore set out to test the hypothesis that the relationship between quantity and quality of ideas, is moderated by the individual differences in Openness to Experience, so that a person with high creative personality may be better able to translate a large quantity of ideas into increasing average creative value.

## Materials and Methods

### Participants

A hundred and fifty four graduate students from the Copenhagen Business School participated in the study. A *post hoc* power analysis using G^∗^Power (Faul et al., 2009) confirmed that our sample size suffices to achieve a statistical power larger than 0.95 for all multiple regression tests reported below, provided that alpha = 0.05 and effect sizes are at least medium (i.e., *f*^2^ = 0.15, Cohen, 1988). The average age of the participants was 22 (range 19–28), with 75 female participants. All participants were randomly recruited from campus and were compensated with a canteen voucher worth approximately 7 Euros. They were all native speakers of Danish. In a Danish context, ethical approval is not required for this type of behavioral non-medical study. Two respondents failed to fill in our measure of creative personality, and were thus excluded from the experiment.

### Psychometric Tests

After signing an informed consent form participants completed a battery of psychometric tests on an online platform developed for the project. The first test was the alternate uses test (AUT) (adapted from Guilford, 1956; Harrington, 1975). In this creative production test participants are presented with a common object and asked to list as many alternative uses they can possibly think of within a 3 min period. Participants were instructed not to include ordinary or unrealistic uses and were given an example object (a paperclip) to illustrate. Ordinary use: hold paper together; unusual use: use as an earring; unrealistic use: fly it to the moon. Participants were given the five objects: Newspaper, Pencil, Towel, Brick, and Shoe. The order of the presented objects was randomized across subjects. For the present experiment creative personality was assessed by the personality trait Openness to Experience (Costa and McCrae, 1992), whereas the other BIG5 dimensions were assessed.

### Coding Procedures

The quantity of the ideas (Fluency) generated on the AUT was determined simply by counting the number of responses, averaged across the 5 objects. The quality of each idea was rated by two trained judges using the judge rating procedure developed by Silvia et al. (2008), which we for the present purposes have labeled “Creative value.” The raters were told to consider three facets in their creativity ratings of the objects: uncommonness, remoteness and cleverness, noting that the strength in one facet can balance weakness in another facet (Silvia et al., 2008). First, each idea was rated for uncommonness (the infrequency of the idea), remoteness (how “far from” a common use), and cleverness (how clever was the idea). Guided by on these three facet ratings each idea was given a final subjective creativity score ranging from 1 (not at all creative) to 5 (highly creative). The subjective creativity score for each response thus constitutes a second coding step and is not merely an automated summing of the facet scores, although creativity and the sum of the facet scores do correlate highly (*r* = 0.836). Each subject was assigned an overall “creative value” score, which is the average score of quality of all ideas produced by an individual. Please note in interpreting the results that creative value should not be mistaken for other common measures used in Equal Odds research (such as the number of hits generated). Each judge rated one half of the total sample of ideas. Inter-rater reliability was assessed by having the two judges rate a sample of the same ideas (39% of the total pool of ideas produced; *n* = 487). Defining the two ratings as in agreement whenever they fell within one point of each other (e.g., Diehl and Stroebe, 1987; Paulus et al., 2011) the two raters agreed in 95.7% of the ratings. Internal consistency between judges, calculated on the raw judge scores was acceptable (ICC two-way, mixed, for consistency =0.68).

## Results

Multiple linear regression analysis was used to develop a model for predicting subject’s average Creative value from their Fluency of idea production (Quantity) and Openness to Experience. We included average idea size (no of characters) as a covariate of no interest. To estimate whether Openness to Experience moderated the relation between average Quantity and average Quality, we used a regression model entered in two levels. Creative value was set as the DV, and (block one) Fluency, Openness to Experience, and idea size as IVs. At block two, the interaction term of Openness to Experience and Fluency was added to test for moderation. The Fluency and Openness to Experience variables were mean-centered. Basic descriptive statistics are shown in Table 1. The model did not indicate evidence of multicollinearity, as evidenced in tolerance scores >0.78 and VIF scores <1.3.

**Table 1:** Descriptive statistics and correlation matrix.

	Mean	*SD*	Quantity	Quality	Personality	Idea size
Quantity (fluency)	9.2	4.4	1.00	0.32***	0.25**	-0.27***
Quality (creative value)	2.3	0.3		1.00	0.41***	0.16
Personality (openness)	119.2	19.2			1.00	0.10
Idea size (no. of characters)	17.6	8.0				1.00
Age	22.0	2.0				


*^∗∗^*p* < 0.01; ^∗∗∗^*p* < 0.001.*

The block one model significantly predicted Creative value, *F*(3,148) = 17.90, *p* < 0.001, with an *R*^2^ of 0.27. Each of the IV’s significantly predicted average Creative value: Fluency (β = 0.305, *p* < 0.001), Openness to Experience (β = 0.314, *p* < 0.001), and idea size (β = 0.224, *p* < 0.003).

The block two model also significantly predicted Creative value, *F*(4,147) = 15.08, *p* < 0.001, with an *R*^2^ of 0.29. Again, each of the IVs’, including the interaction term, significantly predicted Creative value: Fluency (β = 0.256, *p* < 0.002), Openness to Experience (β = 0.328, *p* < 0.001), idea size (β = 0.237, *p* < 0.002), and Fluency X Openness to Experience (β = 0.166, *p* < 0.025). By implication, the difference in *R*^2^ between the two blocks was also significant, *F*(1,147) = 5.13. The regression lines were checked for outliers, and the results remained significant in their absence.

**FIGURE 1:**
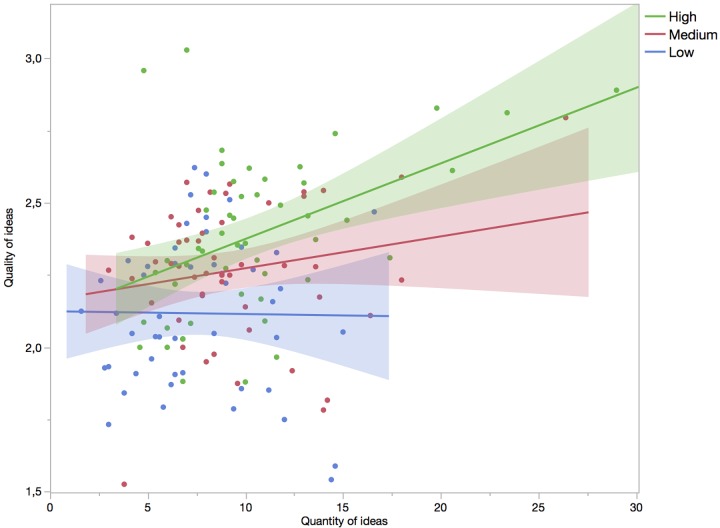
The correlation between the quantity of ideas produced and the rated quality of each idea, which for visualization purposes is calculated separately for each of three levels of openness to experience (high, medium, and low). The color bands each represent the 95% confidence region for the fitted line.

The results are in alignment with past research, and indicate that both Openness to Experience and ideational Fluency directly and independently affect the creative value of ideas. The novel finding here is, however, that Openness to Experience moderates the effect of the quantity (Fluency) of ideas onto their average Creative value. The moderation is illustrated in Figure 1, where the sample, for visualization purposes, is split into three equally sized groups (33rd, 66th percentile) by Openness to Experience scores. It appears as if individuals low on Openness to Experience in effect display a strict version of the Equal-Odds rule, indicating that for this group producing more ideas does not increase average Creative value. But as the level of Openness to Experience increases, the relation of quantity of ideas to average Creative value gradually becomes positive, with a very strong and positive relationship for the group highest in Openness to Experience.

## Discussion

The present results indicate that while quantity does breed quality in creative production, the effect is moderated by individual differences, specifically the personality trait Openness to Experience. Given the assertion in the Equal Odds rule that the relation between idea production and creative hits are characterized as positive, linear, stochastic and constant, then it could be argued that individual average quality of ideas should be constant and unrelated to the individual volume of ideas produced. While this was what we found for individuals low on Openness to Experience, this is not the case across the full spectrum of Openness to Experience. Indeed, with increasing levels of Openness to Experience, the average individual creative value increased sharply with ideational fluency. The results challenge the simple but broadly accepted assertion that *only* quantity drives ideational quality, and underscores the importance of incorporating individual differences in creative personality into models, in this case Openness to Experience. The results do not explain exactly which role Openness to Experience serves in mitigating the relation between quality and quality, but one likely explanation is that openness to new experiences translates into incorporating more variability and novelty into solutions over the course of ideational production, which then is appreciated by judges making creative ratings. This is supported by recent advances in neuroimaging, where it has been documented that individuals high in creativity, reflexively activate more associative memory structures that is linearly predictive of trial-by-trial variability in ideational Fluency, but also that this predictability is higher the higher the creative potential (Friis-Olivarius et al., 2017). As an explanation for the present results this might mean that more creative individuals activate more memory structures, enabling them to better utilize their accumulated knowledge in idea production resulting in more and better ideas.

The present findings also open up a new theoretical avenue in creativity research: the hunt for moderators of the QBQ effect. Further, the results are also of practical relevance for the facilitation of creativity by underscoring the importance of not just encouraging the production of many ideas, but also in considering the personality characteristics of the sample of individuals doing the idea generation. While much past research has focused on increasing informational diversity in teams in order to increase the unshared knowledge that may potentially inform the problem at hand, personality of the team members has typically not been considered important. Further research is needed to examine whether these findings generalize to other types of ideation than the alternative uses test (e.g., to real-world brainstorms), to other types of individual differences than Openness (e.g., using the remote Associates Test), and to examine whether the findings will generalize from individual sessions to group ideation settings.

## Author Contributions

All authors listed have made a substantial, direct and intellectual contribution to the work, and approved it for publication.

## Conflict of Interest Statement

The authors declare that the research was conducted in the absence of any commercial or financial relationships that could be construed as a potential conflict of interest.
